# Graphene Oxide Nanostructures as Nanoplatforms for Delivering Natural Therapeutic Agents: Applications in Cancer Treatment, Bacterial Infections, and Bone Regeneration Medicine

**DOI:** 10.3390/nano13192666

**Published:** 2023-09-28

**Authors:** Khaled AbouAitah, Farzaneh Sabbagh, Beom Soo Kim

**Affiliations:** 1 Department of Chemical Engineering, Chungbuk National University, Cheongju 28644, Republic of Korea; ke.abouaitah@nrc.sci.eg (K.A.); f.sabbagh@chungbuk.ac.kr (F.S.); 2 Medicinal and Aromatic Plants Research Department, Pharmaceutical and Drug Industries Research Institute, National Research Centre (NRC), 33 El-Behouth Street, Dokki, Giza 12622, Egypt

**Keywords:** nanographene oxide, cancer therapy, bacterial infections, bone regeneration medicine, nanoformulations, delivery systems, natural compounds, natural agents

## Abstract

Graphene, fullerenes, diamond, carbon nanotubes, and carbon dots are just a few of the carbon-based nanomaterials that have gained enormous popularity in a variety of scientific disciplines and industrial uses. As a two-dimensional material in the creation of therapeutic delivery systems for many illnesses, nanosized graphene oxide (NGO) is now garnering a large amount of attention among these materials. In addition to other benefits, NGO functions as a drug nanocarrier with remarkable biocompatibility, high pharmaceutical loading capacity, controlled drug release capability, biological imaging efficiency, multifunctional nanoplatform properties, and the power to increase the therapeutic efficacy of loaded agents. Thus, NGO is a perfect nanoplatform for the development of drug delivery systems (DDSs) to both detect and treat a variety of ailments. This review article’s main focus is on investigating surface functionality, drug-loading methods, and drug release patterns designed particularly for smart delivery systems. The paper also examines the relevance of using NGOs to build DDSs and considers prospective uses in the treatment of diseases including cancer, infection by bacteria, and bone regeneration medicine. These factors cover the use of naturally occurring medicinal substances produced from plant-based sources.

## 1. Introduction

Applications for carbon-based nanomaterials of various dimensions may be found in nanotechnology. Diamond, amorphous carbon, and graphite are the three main carbon allotropes found in nature. Additionally, carbon has the extraordinary capacity to hybridize its orbitals in sp, sp^2^, and sp^3^ configurations, producing a variety of allotropes [1]. The abundance of allotropes is closely related to the fact that carbon is the most adaptable substance in the periodic table of elements [2]. The development of nanotechnology has produced a wide variety of carbon-based tiny materials, each with a unique nanostructure designed for a particular use. These include fullerenes, small diamonds, carbon nanocones, carbon nanohorns, single-walled carbon nanotubes (SWCNTs), multi-walled carbon nanotubes (MWCNTs), graphene oxide (GO), and nanosized graphene oxide (NGO) [3,4,5,6,7]. The utilization of carbon-derived nanotechnologies in biomedical applications for nanomedicine and drug delivery systems (DDSs) has accelerated significantly, as indicated by trends throughout time and extensive reviews in more recent years [8,9,10,11]. These nanostructures have remarkable properties, including flexibility, electrical conductivity, mechanical, electrical, and structural variety. They are thus shown to be incredibly well-suited for diagnosing and treating a wide range of illnesses [12,13,14]. It is important to note that graphene is a two-dimensional (2D) material characterized by a uniform, flat monolayer consisting of carbon atoms organized in a hexagonal honeycomb lattice, which is the focus of our specific attention in this study on NGO [15]. Notably, graphene’s unique properties make it an interesting substance on the frontier of 21st-century innovation, rising quickly to prominence in the field of materials research and practical applications [16,17,18]. The synthesis of GO at the micro- and nanoscales has been accomplished using a variety of techniques. Some of these techniques are known to effectively create graphite oxide, a step to creating GO. These techniques include the Hofmann method, Brodie approach, Staudenmaier method, and Hummers’ method, in addition to several variations that developed from these [18]. Due to its extensive use and efficiency, Hummers’ approach has become the standard method for producing graphene oxide nanoparticles [19,20]. The first separation of single-layer graphene from graphite was accomplished in 2004 by University of Manchester scientists Novoselov and Geim [21] using a simple mechanical peeling method. Following this discovery, graphene saw a rise in attention and a variety of uses across several industries [22]. There are several different ways to create GO, including two-layered GO (2 layers), few-layered GO (3–4 layers), multi-layered GO (5–10 layers), and graphite oxide (>10 layers) [23].

**Figure 1:**
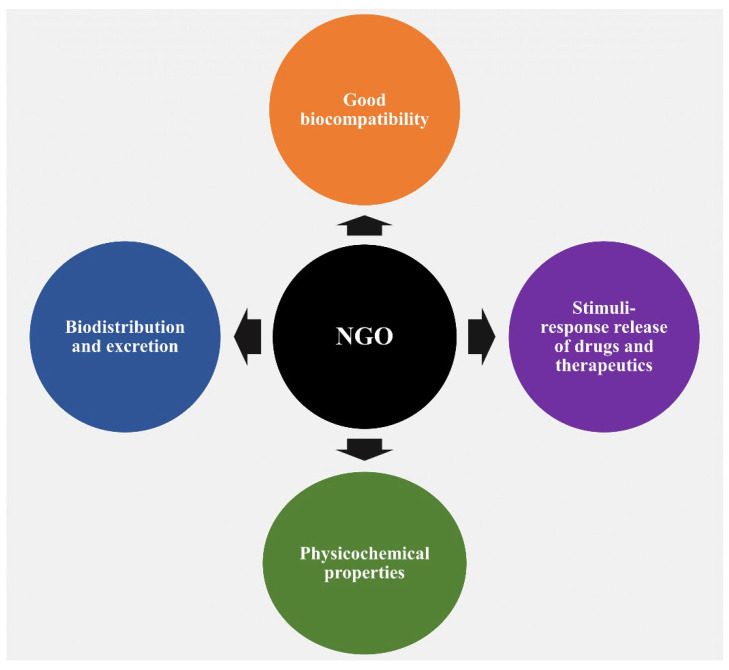
A schematic depiction illustrating the diverse properties of NGOs and their applicability in drug delivery systems, tissue engineering, and biomedical applications.

NGO is increasingly being used in a variety of delivery systems, including drugs, proteins, small therapeutic molecules, and genes, along with diverse biomedical applications including bone regeneration, implants, and more. These applications are in addition to its fundamental properties, which it shares with other carbon-based nanomaterials, including electrical, optical, and thermal characteristics. The numerous characteristics of NGO are mostly responsible for this widespread use. In this article, we discuss a number of reasons that highlight the increased interest in using NGO in pharma and biological contexts (Figure 1).

**Figure 2:**
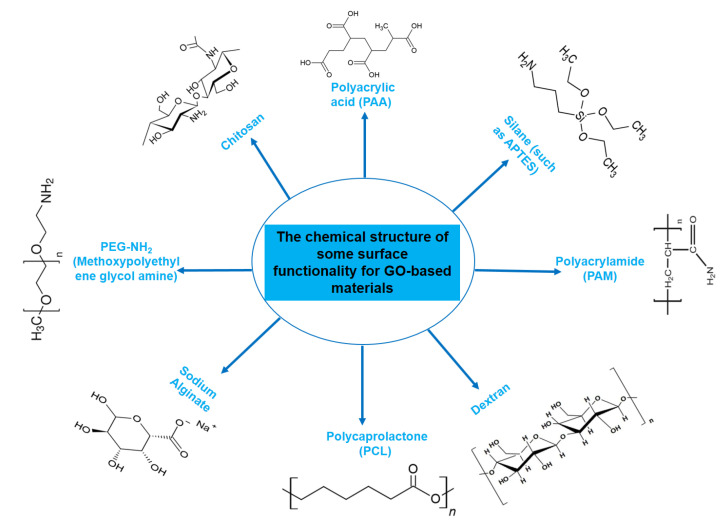
Chemical structures of molecules frequently used for functionalization of GO-based materials. Chemical structures of chitosan [33], polyacrylamide (PAM) [34], dextran [35], and polycaprolactone (PCL) [36]. Polyacrylic acid (PAA), APTES, dextran, and other chemical structures were obtained from chemspider.com, the website of Royal Society of Chemistry (http://www.chemspider.com/Chemical-Structure, accessed on 21 September 2023). PEG-NH_2_ (methoxypolyethylene glycol amine) was taken from the Sigma Aldrich website (https://www.sigmaaldrich.com, accessed on 21 September 2023).

One noteworthy reason for this interest is the observed good biocompatibility of NGOs, which is largely related to dose-dependent toxicity [24,25,26]. The biocompatibility of any nanocarrier should be understood as it is a key factor in determining the best way to include it in DDSs. After administration, graphene, a typical carbon-based nanomaterial, has the capacity to pass through physiological barriers or cellular structures, allowing it to enter the body or cells. It is crucial to note that this process can cause toxicity, both in vitro and in vivo [27]. A wide range of factors, such as various administration routes, entry mechanisms, distribution patterns, excretion pathways, cell uptake behaviors, and particular locations within the biological system, have a significant impact on the degree of toxicity, or conversely, biocompatibility and safety [28,29,30]. Regarding this essential characteristic, Wang et al.’s earlier research thoroughly evaluated the biocompatibility of GO, both in vitro employing human fibroblast cells and in vivo using mice [24]. The results showed that GOs at concentrations below 20 μg/mL exhibited no cellular toxic effects, and the intermediate dose of 0.25 mg showed no detectable harm to mice after cells were exposed to various concentrations for a period of up to 5 days, followed by injection of mice with varying concentrations over a period of 30 days. Notably, different hazardous responses were produced in cellular and animal models at greater dosages and in certain disease stages. In another study conducted by Zhang et al., administration of GO to mice at a dosage of 1 mg/kg of body weight for 14 days did not cause any detectable pathological alterations in the organs evaluated [31]. Furthermore, in a different study, Liao et al. found that aggregated graphene sheets had a stronger cytotoxic effect on human skin fibroblast cells than graphene oxide [25]. Similar to this, Zhang et al. showed that ultrasmall GO nanosheets differ from bigger, randomly sized nanosheets in that they display reduced cytotoxicity while permitting improved cellular uptake [32]. The surface functionalization strategy has recently gained acceptance among scientists as a means of improving the biocompatibility of graphene nanomaterials. NGO-NH_2_ (aminated), NGO-polyacrylamide functionalization (NGO-PAM), NGO-polyacrylic acid functionalization (NGO-PAA), NGO-pegylated functionalization (NGO-PEG), NGO-chitosan, and other modifications are noteworthy among these functionalizations (Figure 2).

In comparison with graphene oxide nanoparticles, these functionalized versions exhibit decreased toxicity [37,38,39,40,41,42,43,44,45]. Kiew et al. briefly summarized the in vitro and in vivo biocompatibility aspects; they noticed that surface functionalization further improves biocompatibility and that NGO displays lower toxicity than micro-sized graphene oxide. This improvement is a result of less material interaction with biological barriers in vitro. Additionally, as compared to their micro-sized counterparts, NGO exhibits higher biocompatibility, and coating the surface helps to prevent accumulation and ameliorate toxic effects in vivo, improving overall biocompatibility [30].

In addition, Bussy et al. have provided a series of recommendations for the use and advancement of graphene and its derivatives, with a view to boosting general safety and reducing the possibility of negative responses in individuals exposed to graphene [46]. These regulations are divided into three main categories: (1) Using small-scale graphene and distinct sheets makes it easier for body macrophages to efficiently internalize and remove graphene from deposition locations. (2) Utilizing hydrophilic, reliable, colloidal dispersions reduces the chance of graphene aggregation within the body and ensures stability for in vivo applications. (3) The use of excretable or chemically modified graphene guarantees that the material is either able to be excreted from the body or is efficiently engineered to decay. Together, these regulations improve safety profiles and reduce the possibility of adverse effects in situations involving human exposure.

According to a review undertaken by Zhang et al. [47], the contact of NGO with plasma membrane and its subsequent translocation across this membrane comprise crucial processes determining the induction of biological effects. The physical and chemical characteristics of NGO, including its size, shape, and surface chemistry, substantially influence the nature of these effects. As a result, it becomes possible to improve transit efficiency while also reducing potential toxicity or negative effects by strategically manipulating these characteristics [47]. A wide range of evaluations on numerous topics, such as biocompatibility, biosafety issues, and contradictions about the impact of graphene nanoparticles, have been offered for a more thorough investigation of this topic [27,30,47,48,49,50,51]. These reviews more deeply examine the complex dynamics that control how graphene nanoparticles interact with biological systems.

The second rationale for utilizing NGOs revolves around their biodistribution and subsequent excretion from the body, which offers insights into potential biomedical applications. Notably, studies on GO sheets that were supplied intravenously and originally had just a few layers (5 nm) showed that these sheets remained in the bodies of living animals for 24 h, mostly localizing in the liver and spleen. Additionally, over 75 percent of the sheets injected into mice were excreted in urine within 24 h of leaving the body, primarily through the kidneys [52]. In a different study, Swiss albino mice were intravenously given (20 mg/kg) non-functionalized, carboxylated, and pegylated nanographene with 2–4 layers (about 100–200 nm) over the course of three months to determine its acute and long-term toxicity [53]. The results showed that no toxicity, organ damage, or substantial modifications were seen despite the continued presence of NGO-PEG inside the liver and spleen tissues throughout the course of the three-month timeframe. These results overwhelmingly point to the material’s potential for biodegradation. However, there are discrepancies in the published data on biodistribution and excretion pathways at the level of animal research. These variances are seen in a variety of organs and are directly related to the specific features of the synthesized NGOs [30,46,51,54,55,56]. This heterogeneity in findings underscores the complex interplay between the properties of the nanomaterials and their interaction with biological systems.

The third rationale for employing NGOs stems from their numerous physicochemical properties, which have been uncovered since their discovery in 2004. These characteristics include a remarkably large surface area, π−π stacking interactions, electrostatic and hydrophobic interactions, streamlined biofunctionalization, efficient water dispersibility, pH-sensitive zeta potential, distinctive intrinsic optical properties, drug-loading capacities, controlled drug release in response to stimuli, applications in photodynamic therapy, and optical imaging functionalities [15,30,51,57]. This multifaceted range of properties positions NGOs as versatile candidates for a wide spectrum of biomedical applications.

The fourth incentive for utilizing NGOs is rooted in their capacity for stimuli-responsive drug and therapeutic release. This material has been extensively used in applications that respond to stimuli. It is noteworthy that several DDSs have been developed with the aid of NGOs to enable the regulated release of medications or therapeutic chemicals in response to internal or external stimuli. These stimuli include variables such as pH, glutathione concentrations, light, magnetic and electric fields, and temperature changes [58,59,60]. The versatility of NGOs in responding to diverse triggers enhances their potential for tailored drug delivery strategies.

The world of nature provides a variety of helpful chemicals originating from numerous living species, such as plants, fungi, and bacteria, when it comes to medicinal agents. Natural products, also known as natural chemicals or secondary metabolites, take on a crucial role in the context of plants. These chemicals, which are referred to as secondary metabolites, are mostly tiny organic molecules with a variety of chemical configurations. Their categorization is frequently dependent on the precise paths they follow throughout the synthesis [61]. Therefore, these natural products can be classified into four major groups: alkaloids (including pyrrolidines, pyrrolizidines, pyridines, tropanes, isoquinolines, indoles, quinolines, and the terpenoids and steroids), phenolic compounds (including phenolic acids, flavonoids, stilbenes, tannins, and lignans), terpenoids (monoterpenes, sesquiterpenes, diterpenes, sesterpenes, and triterpenes), and glucosinolates (sulfur-containing compounds) [62]. In Table 1, several examples of these compounds with their chemical structures are presented. Another division into three major groups includes terpenoids, which are polymeric isoprene derivatives biosynthesized from acetate via the mevalonic acid pathway; phenolics, which contain one or more hydroxylated aromatic rings; and alkaloids, which are nitrogen-containing compounds that are not proteins and are biosynthesized from amino acids [63]. Natural products display a variety of biological and pharmacological actions, making them excellent candidates for the treatment of an array of disorders. These actions include, but are not limited to, a wide range of purposes such as anticancer and antimicrobial activities, and the encouragement of bone formation. The rich spectrum of biological and pharmacological activities exhibited by natural products underscores their potential in developing therapeutic interventions for a variety of ailments [63,64,65,66,67,68,69,70,71,72,73,74,75,76]. Data from the Food and Drug Administration (FDA) emphasize the importance of these natural compounds and highlight their crucial role in the development of therapeutic medications for human use. Notably, almost 40% of the authorized medicinal compounds used in clinical applications have natural product origins or inspiration. It is interesting to note that this tendency is especially noticeable in the area of anticancer therapy, where over 74% of therapeutic agents have their origins in or are inspired by natural substances [70]. Paclitaxel, a popular treatment for breast cancer, serves as an instructive example of how natural compounds may be used to treat cancer. Initially extracted from the bark of *Taxus brevifolia*, paclitaxel has been sold under the trade name Taxol^®^ since 1993. To address the need on a worldwide basis, it is currently manufactured synthetically on a larger scale. Because of this substance’s exceptional anticancer effectiveness, chemotherapy has come to rely heavily on it [77,78]. This is an example of how using natural ingredients may lead to important therapeutic improvements. In fact, as shown in Table 2, natural substances are not only used in medical settings, but are also being studied through clinical studies [70,79]. Table 3 also provides a thorough overview of the factors involved in treatment techniques and lists the benefits and drawbacks of using natural vs. synthetic remedies.

Our main objectives in this review are to investigate surface functioning, drug-loading methods, and approaches to achieving controlled drug release patterns. The focus is on possible pharmaceutical and medical uses, particularly in relation to bacterial infections, cancer, and bone regeneration therapy. Our review’s main focus is the use of DDSs created by NGOs constructed using medicinal compounds derived from plant-derived substances. It is notable that, as far as we are aware, no review paper has yet been published that focuses on the delivery systems designed for natural therapies using NGO materials. Our study seeks to provide useful insight and a thorough grasp of this developing topic by filling this research gap.

## 2. Surface Functionality of NGO

**Figure 3:**
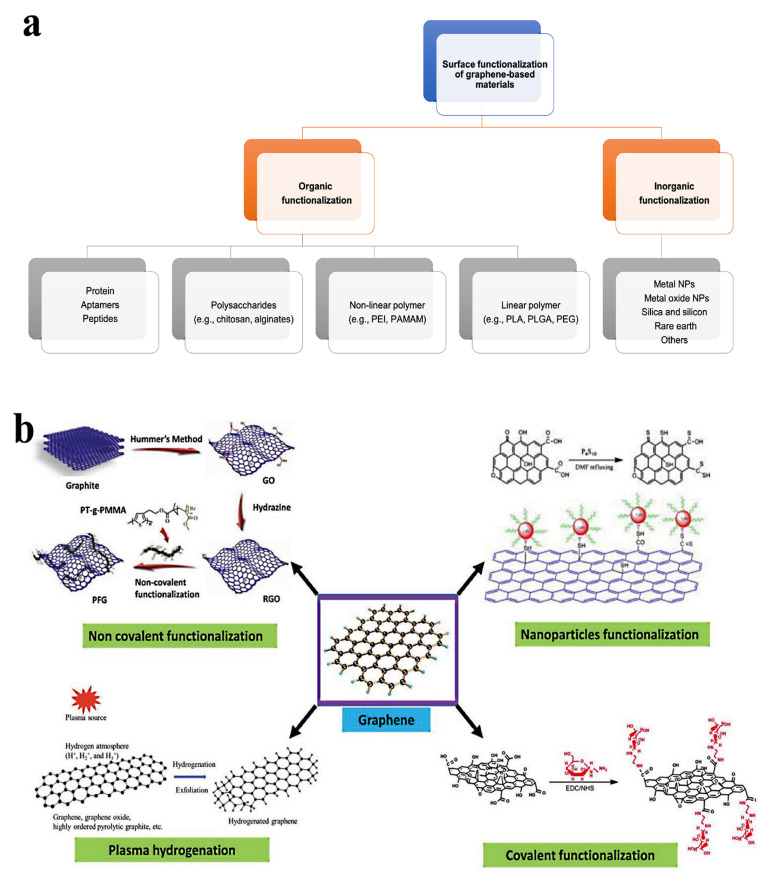
The surface functionalization strategies for GO-based nanomaterial. (**a**) Surface functionalization methods for graphene oxide-based nanostructures: organic and inorganic functionalization. Reproduced from Ref. [86], e-Century Publishing Corporation, an open-access source. (**b**) Surface functionalization approaches for graphene oxide-based nanostructures: non-covalent, covalent, nanoparticles, and plasma hydrogen functionalization. Adapted from Ref. [87]. This is an open-access article distributed under the terms of the Creative Commons CC BY license, published by Advanced Biology, John Wiley and Sons.

**Figure 4:**
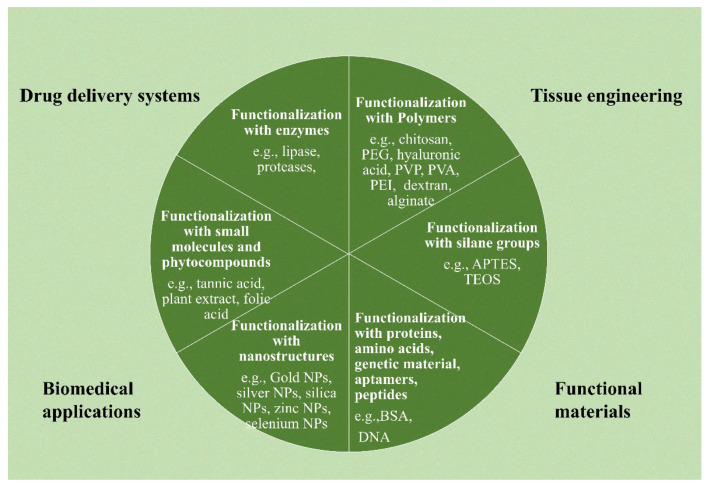
Surface functionalization strategies can be possible for the functionalization of graphene oxide-based nanostructures: functionalization with polymers; with silane functional groups; with proteins, genetic materials, amino acids, peptides, and aptamers; with nanostructures, small molecules, and phyto-compounds; and with enzymes.

Due to its oxygen functions, graphene oxide can be functionalized more easily than other carbon nanomaterials [85]. According to the literature, there are several variations of this functionalization process. For instance, Han et al. [86] divided functionalization techniques for graphene oxide-based materials used in gene and drug delivery into organic and inorganic functionalization, each having distinct subcategories (as illustrated in Figure 3a). Additionally, as shown in Figure 3b, Guo et al. [87] classified the surface functionalization techniques into covalent, non-covalent, plasma hydrogenation, and nanoparticle functionalization. As shown in Figure 4, multiple techniques may be used in the context of NGOs to implement various functionalization methodologies for applications in biomedicine, tissue engineering, drug delivery, and functional material development. This large range of functionalization options illustrates the flexibility and versatility of NGOs for a variety of applications.

**Figure 5:**
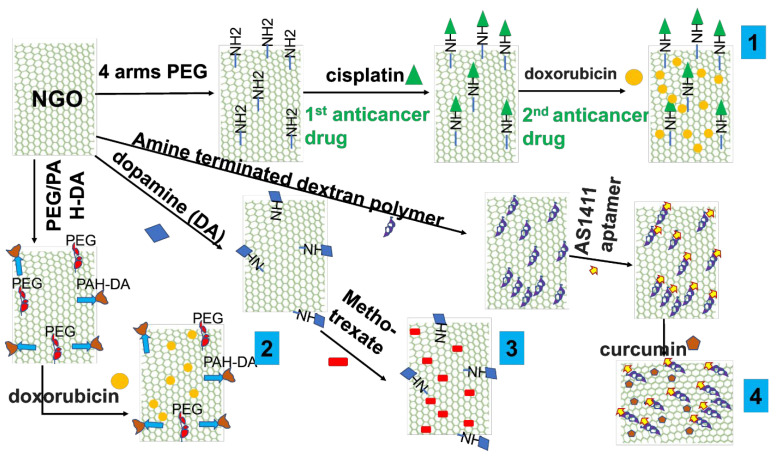
Schematic illustration of feasible functionalization of NGO for cancer delivery designs. (1) Schematic illustration for co-delivery of cisplatin and doxorubicin for cancer therapy [88]. (2) Schematic illustration for combined chemo and photothermal therapy to overcome drug resistance [89]. (3) Schematic illustration for methotrexate anticancer drug [90]. (4) Schematic illustration for targeted delivery system by aptamer with curcumin natural model anticancer therapy [91].

The possibility of functionalizing NGOs to create medicine delivery systems is demonstrated in this section with several examples. For instance, Pei et al. [88] showed a co-delivery strategy for two anticancer medicines using a PEGylated-functionalized NGO. In the first stage, NGO containing carboxylic acid groups underwent functionalization with four-armed PEG-NH_2_ by creating a covalent amide connection. The PEGylated nanoparticles were then coupled with N-hydroxysuccinimide (NHS) and 1-ethyl-3-(3-dimethylaminopropyl)carbodiimide (EDC) to deliver cisplatin. Following that, more doxorubicin was loaded, which was made possible by a stacking interaction (Figure 5-1).

A smart pH-responsive drug delivery system using NGO was also reported by Feng et al. [89] in order to combat drug resistance using a combination of chemo and photothermal treatment. In this design, PEG and positively charged poly(allylamine hydrochloride) (PAH) were added to NGO to modify it. 2,3-Dimethylmaleic anhydride (DA) was used to further modify PAH (Figure 5-2). The doxorubicin-loaded functionalized NGO then demonstrated a pH-responsive capacity to cause cell death in both drug-sensitive MCF-7/ADR cells and wild-type MCF-7/WT cells. Another noteworthy example is the work of Masoudipour et al. [90], who constructed a dopamine-conjugated NGO by EDC/NHS coupling chemistry. The antitumor medication methotrexate was delivered using this combination as a nanocarrier. The delivery method provided a targeted and tailored approach for drug administration by specifically targeting the DA receptor in human breast cancer cell lines that were positive (Figure 5-3). Furthermore, Alibolandi et al. [91] reported covalently functionalized NGO with an amine-terminated dextran (EDA-DEX) polymer using EDC reagent. Then, the functionalized NGO was coupled to the AS1411 aptamer and curcumin was loaded with the assistance of π−π stacking interactions. When tested against the cancer cells 4T1 and MCF-7, this complete design demonstrated noteworthy results, including effective cellular uptake and strong anticancer effects (Figure 5-4).

In fact, the growing problem of organisms’ resistance to conventional antibiotics has become a major obstacle. To overcome this issue, researchers are now looking into NGOs’ use in medication delivery systems. For instance, Erdal et al. [92] described a process that included grafting porous poly(ε-caprolactone) (PCL) scaffolds with cellulose-derived NGO on their surfaces. The localized delivery of the antibiotic ciprofloxacin was the goal of this concept. Meanwhile, Lu et al. [93] demonstrated the potential of GO nanosheets with amine functionalization for powerful antibacterial applications. Polyethylenimine (PEI)-functionalized NGO was designed to transport the antisense walR plasmid in a work by Wu et al. [94]. The proposed method successfully inhibited *Enterococcus faecalis* bacteria, demonstrating the promise of functionalized NGOs for focused antimicrobial therapies. Another noteworthy instance involves the creation of a strategy for photodynamic therapy to treat microorganisms. In this instance, curcumin-functionalized graphene quantum dots were used [95]. *Pseudomonas aeruginosa*, methicillin-resistant *Staphylococcus aureus* (MRSA), *Escherichia coli*, and *Candida albicans* were only some of the pathogens that this system showed improved antimicrobial activity against. It is important to note that materials based on NGO, even those with very small dimensions, such as quantum dots, have displayed astounding efficiency against viruses. This relevance is illustrated by a study that showed functionalized graphene quantum dots and boric acid have the potential to inhibit the highly pathogenic human coronavirus (HCoV-229E) by impairing both viral entrance and reproduction processes [96]. In a previous study, Kim et al. [97] described a delivery method intended particularly to target and control hepatitis C. This strategy made use of NGO conjugated with DNAzyme (deoxyribose) and loaded onto its surface. GO materials provide specific benefits for use in bone regeneration therapy in addition to having superior electrical and mechanical characteristics in comparison to other materials. These benefits are primarily attributable to their innate capacity to support various functionalities. These include giving simple access to facilities for fabrication, the development of several composites, and the construction of scaffolds, all of which contribute to their enormous potential in the field of biomedicine [98,99]. The use of the NGO matrix is central to three widely used approaches in the field of bone regeneration medicine: coatings, controlled release of medicines, and composites [99]. For example, electrospun scaffolds were produced by integrating graphene oxide dots into polymers (such as polylactic acid (PLA) or PCL) during the electrospinning process. This novel strategy is intended to improve mechanical qualities, as well as encourage osteobioactivity [100]. In another instance, researchers added GO nanosheets to methacrylated gelatin (GelMA)-based scaffolds to help human mesenchymal stem cells produce bone [101]. Additionally, it was shown that functionalized scaffolds with NGO on the surface allowed for effective ciprofloxacin loading and release for a longer period of time depending on the predominance of π−π interactions between the antibiotic and NGO; thus, using NGO has led to enhanced compressive strength and bioactivity of the scaffolds [92]. Furthermore, the release of the medication displayed a regulated pattern that was significantly impacted by pH levels when doxorubicin was incorporated into polyethylene oxide/chitosan/GO nanofibrous scaffolds. A share of 40% of the drug was released within 72 h, indicating a substantial pH dependence in the release kinetics [102].

## 3. Drug Loading and Drug Release Profile

### 3.1. Drug-Loading Strategies

#### 3.1.1. Drug Solubility

Due to their effective drug-loading capacities, GO and its derivatives exhibit possibilities in the field of medication delivery. However, it is difficult to successfully load medicines with high solubility onto these materials [103]. The two-dimensional aromatic surface of GO and its derivatives makes them ideal for adsorbing pharmaceuticals that have poor solubility using methods such π−π stacking and hydrophobic interactions [104]. An advantageous aspect of utilizing GO-based materials in controlled drug release is their capacity to pass through cell membranes, coupled with their substantial specific surface area [105]. This feature opens up a wide range of possible connections with tailored medication delivery systems. Consequently, a workable method includes releasing medications that have been placed on GO sheets by putting them in biological fluids. The hydroxyl and epoxy groups on GO can form hydrogen bonds with other molecules, while the carboxyl group gives the surface of the compound a negative charge. Because of this quality, GO-based compounds are easily dispersed in polar solvents including water. By adding polymers and other nanoparticles to the surfaces of graphene nanoparticles and their derivatives, one can increase their effectiveness in DDSs [106]. Loading drugs with significant solubility, in particular strong hydrophilic agents, presents challenges due to the lack of robust interactions [103]. This limitation severely limits the possibility of using graphene and its analogs as carriers for highly soluble water-based medications without the use of surface functionalization techniques.

#### 3.1.2. Drug-Loading Approaches

**Figure 6:**
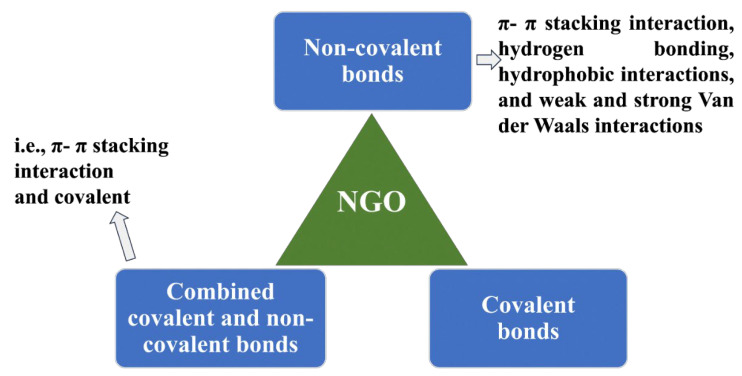
Possible loading approaches of drugs/therapeutic molecules onto NGO and GO-based materials. There are three approaches: non-covalent bonds (π−π stacking interaction, hydrogen bonding, hydrophobic interactions, and weak and strong van der Waals interactions), covalent bonds (such as amino, amide bonding), and combined non-covalent and covalent bonds (π−π stacking interaction and amino covalent bonds) [15,88,111,115,116].

The technique of incorporating pharmaceutical substances or active ingredients (APIs) into a carrier matrix, such as nanoparticles, microparticles, liposomes, or implants, with the goal of delivering them to specific areas inside the body is known as drug loading. For drug loading, a variety of methods are used, including physical encapsulation, co-precipitation, adsorption, coating, solvent evaporation/extraction, and supercritical fluid technology. The above processes make it possible to provide therapeutic ingredients in a focused and regulated manner. The primary objective of incorporating a drug into a carrier material such as carbon, NGO, or mesoporous silica nanoparticles is to maintain the therapeutic agent within the carrier and facilitate its delivery to a particular target site where its therapeutic effects are required. As it affects fundamental characteristics such as loading capacity, the interaction between therapeutic molecules (such as drugs) and NGO is vital in this context. The pharmacological substances must possess these characteristics in order to achieve regulated release patterns and elicit the appropriate therapeutic response. Currently, methods such as molecular dynamics simulation (MDS) are used to investigate how the drug interacts with the carrier substance (such as NGO). Understanding and improving the drug-loading process requires an understanding of binding energies, hydrogen bonds, and dynamic behavior, all of which are rendered possible by MDS. For instance, MDS can shed light on the most efficient ways to load drugs onto NGO when evaluating a medication such as mitoxantrone [107]. It is crucial to remember that GO, which may be used to load drugs, is made up of layers that are formed of six-atom rings organized in a honeycomb structure. This makes GO a genuine planar aromatic macromolecule [17,108]. The basal plane of GO sheets is rich in hydroxyl and epoxy groups, whereas the edges are abundant with carboxyl functional groups [109]. The distinctive functional groups and distinctive hexagonal carbon structural composition contribute to the diverse surface chemistry of GO. Covalent and non-covalent bonds can be created because of this chemistry [110]. The loading technique for pharmaceuticals with NGO may be divided into three main categories: non-covalent bonds, covalent bonds, and mixtures of these two methods (Figure 6). For a wide range of therapeutic substances, including drugs and genetic materials, the non-covalent method of drug loading onto NGO/GO materials has been thoroughly investigated. These studies show that simple physisorption processes, such as π−π stacking (which is thought to exert a considerable force on these materials) or hydrophobic contacts, are the main drug-loading mechanisms that are used to attach drugs to NGO/GO materials. These interactions help to significantly adsorb pharmaceuticals onto GO-based materials, which leads to large drug-loading capabilities [15,111]. The presence of free π electrons on GO’s surface facilitates distinctive interactions with molecules [112]. Drugs and therapeutics can either be water-soluble or hydrophobic (which is a substantial class). Therefore, the free electrons in GO provide a hydrophobic area that is crucial for loading hydrophobic therapeutics through van der Waals forces [113]. Additionally, interactions between medicines and NGO that form hydrogen bonds allow for the process of drug loading [107]. These many interactions, which involve both hydrogen bonds and hydrophobic forces, highlight how adaptable GO and its derivatives are as efficient carriers for a variety of therapeutic treatments. In fact, the loading process is mostly carried out by the adsorption of drugs or other therapeutic molecules onto NGO/GO, which is fueled by a variety of interactions. These interactions include weak and strong van der Waals forces, hydrogen bonds, hydrophobic contacts, and π−π stacking interactions. Together, these pressures make it easier to successfully include pharmaceuticals in NGO/GO carriers. It is also important to note that the covalent bonding strategy is a successful method for medication loading. Between medicines and GO, covalent bonds can develop, offering solid and reliable adhesion [88,114]. As an example, branching polyethyleneimine and NGO-functionalized PEG were used as carriers to covalently load the chemotherapy drug cisplatin [115]. Another work by Wei Zhuang et al. demonstrates a dual strategy using both adsorption and covalent attachment for loading the cancer-targeting medication paclitaxel (PTX) onto functionalized NGO [116]. NGO is an excellent vehicle for combination therapy in the treatment of illness because of its large specific surface area, which allows for a substantial loading capacity. In a work by Pei et al. [88], a novel application is shown where two anticancer medications are coupled on NGO-PEG using a covalent approach. NGO-PEG-Pt is created by conjugating the first medication, cisplatin (Pt), through an amino-bonding process. The second drug, doxorubicin (DOX), was then loaded by a non-covalent method utilizing π−π interactions, creating the dual drug delivery system NGO-PEG-Pt/Dox.

### 3.2. Release from Nanographene Oxide

When a drug is contained inside a nanographene oxide, its release is controlled by the rate at which the drug is released from the graphene oxide [117]. Drug solubility, drug dispersion inside the nanocarrier, bonding interactions, release rate, coatings, and other variables are among the many elements that influence the release of pharmaceuticals. The final profile of drug release from the carrier system is greatly influenced by these variables taken together. 

#### Triggering Drug Release from Nanographene Oxide

Materials based on NGO/GO provide a variety of options for producing flexible drug release effects. Both internal and external cues are used to exercise control over and maintain therapeutic advantages from these effects [15,59]. Drug release from NGO-based materials can be governed using a variety of stimulus-responsive release methods. Each of these systems contributes to tailored and controlled drug release patterns, and they may be designated as internal, external, or mixed effects (Table 4).

A large number of research studies conducted in the framework of NGOs have focused on cancer therapy, including the use of both natural and approved chemotherapy drugs. A collection of research results from diverse studies is shown in Table 5 and Table 6, emphasizing the possibility of drug loading and controlled release made possible by NGO-based systems.

The drug release profiles that are seen in research involving NGOs show signs of controlled and sustained drug release. This has significant implications since it can improve patient compliance by delivering a consistent therapeutic dose and tailoring the therapy’s effects. These positive research findings have significant potential for the creation of more effective and customized therapies. As a result, the delivery of drugs is becoming safer and more efficient in the field of medicine.

Even though these results are motivating, it is vital to recognize that further research and clinical trials are required. Regarding NGOs, these next initiatives are crucial for properly understanding the long-term safety, general effectiveness, and possible uses of synthetic drugs. These studies will advance our knowledge of the therapeutic applications of these cutting-edge drug delivery technologies for the treatment of different diseases. This extensive research will ultimately open the door for the appropriate and knowledgeable integration of NGO-based remedies into medical practice.

## 4. Nanographene Oxide for Natural Anticancer Therapy

**Figure 7:**
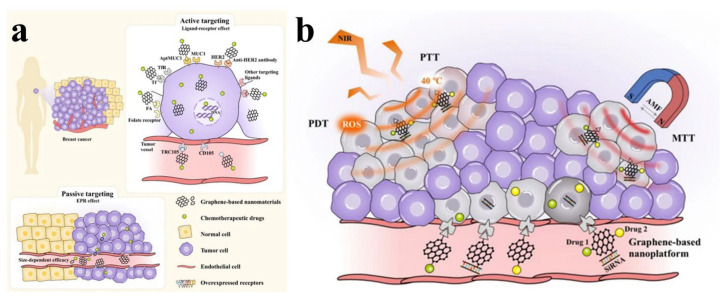
The schematic presentation shows the active and passive targeting based on graphene-based nanomaterials. (**a**) The active site targeting ligands displays the direct attachment with many site-specific receptors, overexpressing on the surface of cancer cells (in this case breast cancer) or tumor vessels, and the targeting effects through receptor-mediated endocytosis and the enhanced permeability and retention (EPR) effect. (**b**) A multifunctional platform established by graphene oxide-based nanomaterials, showing the ability to simultaneously load many therapeutics (drugs and genes), and allow the photothermal therapy (PTT), photodynamic therapy (PDT), magnetothermal therapy (MTT), alternating magnetic field (AMF), and generation of reactive oxygen species (ROS) at the same time. Reproduced from [144], an open-access article, published under a Creative Commons Attribution 4.0 International License, Springer Nature.

Recent studies have emphasized a number of beneficial outcomes that result from the use of graphene-based materials in anticancer applications. These processes result in a variety of outcomes, including the induction of apoptosis for anticancer effects, acting as critical nanocarriers that promote cellular uptake and internal release within cells and tissues, and enabling the transport of specific molecules for uses such as imaging, photodynamic/photothermal therapy, and targeted site delivery (Figure 7) [143,144]. Here, we give a summary of the achieved active targeting ligands used in the development of targeted delivery systems using GO nanomaterials. Peptides, small molecules (such as folic acid, a frequently utilized ligand), proteins, and antibodies are the available ligands for active cancer targeting through non-covalent and covalent bonding (Table 7). The delivery of natural medicines through NGO is a growing area of research interest. Although there are currently very few studies that have reported the use of natural medicinal agents via NGO, it is hoped that more thorough investigations and attempts in this regard will be launched in the future. 

Curcumin, a natural polyphenol produced from the rhizomes of turmeric (*Curcuma longa*), has in fact shown extraordinary anticancer properties. An aptamer was used to decorate a dextran-functionalized NGO to actively target cancer cells in research by Alibolandi et al. [91]. Dextran, a biocompatible polymer, was added covalently to the surface of NGO sheets as part of the design. The AS1411 aptamer was also conjugated to this dextran-modified NGO to enable active site targeting. Following that, the system was filled with curcumin thanks to stacking interactions. The high loading capacity of 29 wt% produced by this method highlights how well drugs are loaded onto NGO. The proposed nanoconstruction design effectively internalized into 4T1 and MCF-7 cells, two types of cancer cells that overexpress nucleolin. The increased internalization resulted in noticeably stronger cytotoxic effects. The potential for developing cutting-edge drug delivery systems that take advantage of natural substances and nanomaterials for improved therapeutic outcomes in cancer treatment is demonstrated by the combination of the targeting strategy using aptamer and the loading of curcumin onto the functionalized NGO. A nanoformulation including GO−gold nanoparticles−curcumin was also prepared by Al-Ani et al. [153]. In vitro testing of this nanoformulation was conducted on colon cancer cells. Based on the study’s findings, this formulation promoted the transport of curcumin into cells and caused cytotoxicity in HT-29 and SW-948 colon cancer cell lines via activating apoptosis through redox. This shows the possibility of integrating graphene-based materials such as GO, gold nanoparticles, and curcumin to produce efficient nanoformulations with the capacity to target and elicit devastating effects on certain cancer cell types. A significant advancement was made possible by the establishment of a dual nanoformulation that combines the well-known anticancer drug doxorubicin with the natural anticancer ingredient curcumin. The main objectives of this formulation were to reduce the adverse effects of doxorubicin while concurrently increasing its therapeutic effectiveness [154]. The AGS human gastric cancer cell line, the PC3 prostate cancer cell line, and the A2780 ovarian cancer cell line were among the cancer cell lines used by the researchers to assess the anticancer properties of this dual formulation. The results of the experiment revealed numerous important discoveries. Both doxorubicin and curcumin can be transported by nanoformulation because of their high drug-loading capacities. Additionally, the medicines’ release patterns were pH-dependent, with the highest release taking place at pH 5.5. Contrary to the other cell lines examined, PC3 cells showed higher sensitivity. Due to the fact that only a portion of the loaded drugs were released, the researchers reached the conclusion that using photodynamic therapy may improve the therapeutic effects even more. This approach shows the possibility of integrating many therapeutic compounds into a single nanoformulation in order to maximize efficacy and reduce side effects. The natural flavonoid quercetin, known for its high antioxidant qualities and well-documented anticancer effects, has drawn considerable interest in the field of medication administration. Researchers are exploring its possible use in various drug delivery systems because of its unique features and potential health advantages. In a study on the delivery of quercetin, 0.4 µm carboxylated-GO sheets were utilized as nanocarriers to deliver quercetin to PC3 prostate cancer cells. This approach demonstrated regulated release mechanisms that were affected by both pH sensitivity and temperature. High cellular internalization, controlled release profile, and efficient cytotoxicity against the intended cancer cells were among the significant properties of the nanoformulation [155]. Likewise, Tiwari et al. [156] developed a co-delivery system. In this system, GO functionalized with polyvinylpyrrolidone polymer was used to produce a nanocarrier that contained both the antioxidant quercetin and the cancer fighter gefitinib. The goal of this hybrid nanoformulation was to give ovarian cancer cells a synergistic boost. According to the findings, the co-delivery technology dramatically increased toxicity when compared to administering each drug individually and free natural components. Comparing PA-1 ovarian cancer cells to IOSE-364 normal ovarian epithelial cells made this enhanced cytotoxicity clear. Protocatechuic acid (PCA) and chlorogenic acid (CA) were combined in a delivery nanoformulation by Buskaran et al. [157]. A PEG-functionalized NGO was used in the formulation approach for the delivery system. EDC/NHS coupling chemistry was used during the synthesis to join the carboxylic acid groups of NGO with those of PEG4000 to create PEG-NGO. Then, via adsorption, this PEG-NGO was employed as a carrier to encapsulate both PCA and CA. Folic acid was then added to the resulting functionalized material, creating a folic acid-modified NGO that enhanced the active targeting of cancer cells. To evaluate cellular absorption, the researchers also created a FITC-labeled folic acid-functionalized NGO. In the nanoformulation, PCA and CA made up around 23.82% and 19.55%, respectively. Notably, after approximately 120 and 130 h, respectively, the release of PCA and CA appeared to be completed. Changes in pH (7.4 and 4.8) affected these release patterns. The researchers highlighted the impact of these parameters on release kinetics by attributing the variations in drug release to factors such as drug arrangement on NGO nanocarriers and drug size. Studies on cellular absorption employing FITC-labeled folic acid-functionalized NGO demonstrated clear uptake in HepG2 hepatocellular cancer cells. Additionally, as compared to separate substances and free natural agents, the combined nanoformulation demonstrated notable cytotoxicity. The computed IC_50_ values for PCA and CA were 40.78 ± 1.92 g/mL and 43.61 ± 1.74 µg/mL, respectively. Further examination of the cell death pathway revealed that HepG2 cells treated with the developed nanosystem induced late apoptosis, G2/M phase cell cycle arrest, depolarized mitochondrial membrane potential, and enhanced generation of reactive oxygen species. Scientists designed an actively targeted nanoformulation for chemo-photothermal synergistic cancer treatment using GO nanosheets and a berberine derivative [131]. The suggested strategy attempted to utilize both the near-infrared (NIR) laser application and the acidic microenvironment of the tumor for dual-responsive medication release. Through an amide reaction, S1411 aptamers were attached to the surface of GO nanosheets to enable active cancer targeting. The endocytosis of the AS1411-GO nanosheets into cancer cells was considerably aided by the conjugation of AS1411 aptamers. The AS1411-conjugated nanosheets were then covered with a berberine 9-O-pyrazole alkyl derivative (as an anticancer drug) via conjugate π−π stacking. The loading capacity of 68.3% was remarkable for this technique. Release experiments were carried out under simulated physiologically normal settings (pH 7.4) and under simulated tumor-acidic conditions (pH 5.3). The findings showed a larger cumulative release at the acidic pH (18%) compared to the physiological pH (12%), indicating a more regulated circulatory release pattern and a faster release in the acidic tumor environment. Notably, the cumulative release effect was much improved when laser radiation was used. An important goal was active targeting, and the AS1411-GO/B3 nanoformulation showed higher accumulation in tumor tissues, especially in A549 cells. Additionally, after being exposed to 128 µg/mL of the nanoformulation, the viability of A549 cells dropped to 81%. The therapeutic response was further enhanced by the addition of laser treatment, leading to higher cytotoxicity and more prominent cell-killing effects. This work serves as an example of the possibility of utilizing sophisticated nanomaterials, such as GO, in conjunction with exact targeting techniques and synergistic therapeutic mechanisms to obtain improved cancer therapy results. GO nanoscrolls were used as carriers for administering gallic acid in a study by Sontakke et al. [158] According to the study’s findings, the pH factor had an impact on how much gallic acid was loaded onto and released from the nanoscrolls. Comparing the A549 lung cancer cell line to the HEK293 healthy cell line, the developed GONS-GA system showed increased toxicity. This approach may be able to efficiently target and destroy cancer cells while preserving healthy cells, according to the differential cytotoxicity. In another study [141], *Juniperus squamata* root essential oil was loaded onto polyvinylpyrrolidone-functionalized GO (GO-PVP) through π−π and hydrophobic interactions. This nanoformulation improved anticancer efficacy, especially against MDA-MB-231 human breast cancer cells.

The accumulated evidence highlights the possibility of using NGO or GO nanoparticles in combination with naturally occurring substances generated from plants for anticancer applications across diverse cancer types. This strategy provides a promising way to increase the therapeutic benefits of cancer-fighting medicines and specifically target cancer cells. The interaction of natural products with nanomaterials creates new opportunities for innovative cancer therapy. Several technological considerations are advised for the development of these smart delivery systems for natural medicinal compounds, which are presented in Table 8.

## 5. Antibacterial Delivery Systems

Nanomaterials based on graphene, such as GO, reduced graphene oxide (rGO), and their composites with metals, metal oxides, and polymers, show promise as antibacterial agents. Because of their antibacterial action, this has attracted a lot of attention from researchers who are investigating their potential as active therapeutic nanomedicine platforms against pathogenic microorganisms [159]. These materials’ potential to fight infectious disorders, especially those brought on by bacteria resistant to many drugs, is being studied [160]. Several crucial considerations must be made while exploring the use of graphene-based nanomaterials as nano-antimicrobial therapeutics. Enhancing effectiveness and lowering possible toxicity require surface modification and functionalization. It is feasible to maximize these nanoparticles’ antibacterial effectiveness while reducing any negative consequences by adding inorganic nanostructures, proteins, or polymers to their surfaces [161]. In previous research, the antibacterial potential of GO was shown to be effective against a number of bacterial species, including *Klebsiella pneumoniae*, *E. coli*, and *Pseudomonas aeruginosa* [162]. A concentration-dependent pattern of the antibacterial actions was seen. In particular, this study discovered that GO was especially successful in eliminating *Klebsiella pneumoniae*. A 250.0 µg/mL GO therapy resulted in an in vitro eradication rate of more than 95%. These results are important not just in vitro but potentially have implications in vivo. These results demonstrate that GO itself is an appropriate material with an antimicrobial function. The capacity of GO to physically damage bacterial membranes is what gives it its first antibacterial capabilities [163].

It has been progressively reported that the NGO’s antibacterial activity is highly promising in the biomedical field, and some studies have shown possible antibacterial mechanisms. In a study by Robb et al. [164], GO antibacterial mechanisms were reported to be unclear, but three hypotheses were presented about the aspects that contribute to antibacterial activity: (i) the edges of the 2D sheets dissolve and destroy the bacterial membrane; (ii) the sheets cover the cell, preventing access to essential nutrients; and (iii) GO results in metabolic oxidative damage to the cells. In their experiments, they indicated that NGO significantly inhibits the development of *E. coli*, but the micrometer-sized GO exhibits no significantly inhibited growth, illustrating the antibacterial efficacy dependent on size features. Additionally, when lipid membrane vesicles are exposed to GO or NGO, a change in lipid composition and tension is made possible. Pulingam et al. revealed the differences in interactions between GO and Gram-positive and Gram-negative bacteria [165]. When GO interacts with bacteria, especially when concentrations rise, bacteria lose membrane integrity by increasing the release of lactose dehydrogenase (LDH) into the reaction medium. The surface morphology of bacterial culture-treated GO demonstrates apparent differences in the GO mechanism of action against Gram-positive and Gram-negative bacteria, where cell entrapment was primarily observed for Gram-positive *S. aureus* and *Enterococcus faecalis*, but membrane disruption was found for Gram-negative *E. coli* and *Pseudomonas aeruginosa*, which is related to the physical contact. By means of ATR-FTIR analysis, the results reveal that treated bacterial cells display changes in fatty acids, amide I and amide II of proteins, peptides, and amino acid regions compared to untreated bacterial cells. In an earlier study by Tu et al. [166], they demonstrated experimentally and theoretically that GO and NGO sheets induce the degradation of the inner and outer cell membranes of *E. coli*, as well as reduce their growth viability. They demonstrated that the process occurs in three general steps using TEM observations. By using molecular dynamics simulations, they were able to identify the precise atomic details involved. They demonstrated that NGO can enter cell membranes and remove significant amounts of phospholipids from them due to the powerful dispersion interactions between nanosheets and lipid molecules. Therefore, disruptive extraction enables nanosheets’ potential mechanisms for cytotoxicity and antibacterial actions against bacteria. Similar to this, Mao et al. [167] investigated the processes behind the cytotoxicity and antibacterial activity of GO in stimulating theoretical and experimental methods. Through theoretical research, they showed the cellular interaction of nanosheets with different levels of oxidation. Four typical states of nanosheet−membrane interactions were seen based on the degree of oxidation and size of graphene nanosheets: superstructures made of sandwiched graphene, graphene that adheres to the membrane, graphene that is spread out over the membrane, and hemispheric vesicle structures. Intriguingly, these stages were found in the experimental data. Importantly, their studies revealed that graphene nanosheets with a higher degree of oxidation may cause a greater degree of irregular membrane disruption, damaging the membrane’s integrity. This is due to these nanosheets, especially when they have increased edge length. As a result of cellular internalization and the cytotoxicity and nanosheets with bacteria, these actions aid in realizing the main antibacterial cytotoxicity actions.

GO has a number of benefits over other commonly utilized nanomaterials when it comes to manufacturing antibacterial drug delivery systems and performing as an antibacterial [161]. The following characteristics best describe these benefits: (1) physical and chemical oxidation has an impact on GO’s antibacterial mechanism, which reduces the resistance of many bacteria; (2) low-concentration doses of GO have a mild cytotoxic effect on mammalian cells; and (3) GO is easier to process, can be produced in large quantities, and is less expensive to produce than other carbon nanomaterials [168,169]. Here we discus some examples showing the advantages of GO-based material delivery systems for drug-resistant bacteria. To treat bacteria that are resistant to antibiotics, near-infrared (NIR) photothermal antibacterial materials based on NGOs were developed. These materials have the biocompatibility property of bovine serum albumin (BSA) and photothermal molecules of aggregation-induced emission fluorogen (AIEgen) [170]. In comparison to single-model phototherapy, the results demonstrate a superior antibacterial effect (>99%) against amoxicillin-resistant *E. coli* and *S. aureus*, making this delivery system a desirable choice for tracking antibiotic resistance in bacteria. In order to treat *Porphyromonas gingivalis* using antimicrobial photodynamic treatment, Pourhajibagher et al. [171] developed and assessed a targeted biotheragnostic system based on DNA-aptamer-NGO. As demonstrated by flow cytometry, they reached the conclusion that the proposed system binds specifically to *P. gingivalis*, and that PDT exposure causes photoactivation and the production of ROS, which greatly reduce *P. gingivalis* numbers. In addition, the DNA-aptamer-NGO-mediated photodynamics display a significant reduction in the biofilms and metabolic activity of bacteria as compared to the control group. When treated bacteria were exposed to radiation, a slight rise in the number of apoptotic cells and significant changes in the expression of specific genes (related to bacterial biofilm formation and response to oxidative stress) occurred at the molecular level. For vancomycin-resistant *Enterococcus* (VRE), a hybrid nanocomposite using GO consisting of vancomycin and photosensitizer phthalocyanine was made via π–π stacking interactions to achieve a phototherapy-based combination strategy [172]. Treating bacteria with this delivery system and application of photothermal and photodynamic treatment result in almost 2–3 logs of bacterial reduction when tested in vitro. The treatment also exhibits infection regression on VRE-infected BALB/C mice, along with accelerating wound healing and negligible damage to normal cells and major organs.

Regarding natural delivery systems, a smart medicine delivery system was developed employing NGO-capped PEG loaded with *Nigella sativa* seed extract [173]. The antibacterial efficacy against *E. coli* and *S. aureus* was examined. Investigation into the antibacterial mode of action revealed that the GO-based system efficiently penetrated bacterial cytoplasmic and nucleic acid membranes. As a result, the integrity of the cell wall was damaged, nucleic acids were harmed, and the permeability of the cell wall increased. In another study, the antibacterial efficacy of a composite made of curcumin and GO against methicillin-resistant *S. aureus* (MRSA) was examined [163]. The results demonstrated this composite’s effective antibacterial ability against MRSA, with activity at doses of less than 2 µg/mL. The fact that this antibacterial activity was attained with no fibroblast damage is significant since it emphasizes the antibacterial action’s selectivity and suggests safe use. Yan et al. [174] focused on creating an efficient antibacterial composite nanomaterial, which resulted in an excellent antimicrobial composite nanomaterial. They accomplished it through π−π stacking interactions to load polydopamine-curcumin onto the surface of GO. This nanocomposite system showed excellent promise for photodynamic treatment in terms of its antibacterial activities, resulting in an incredible decrease in Gram-positive *S. aureus* by four orders of magnitude and an excellent bactericidal rate of 99.99%. This effectiveness was directly related to the production of ROS brought on by white light irradiation (405–780 nm). Bacterial outer membranes were shattered and bacterial cells were deformed as a result of the ROS. This work emphasizes the significance of integrating polydopamine-curcumin with GO to create a viable photodynamic antibacterial material. In a study by Cacaci et al. [175], curcumin was used to formulate an antibacterial strategy that targeted the biofilm formation of *Candida parapsilosis* on medical equipment. Healthcare-associated infections are complicated by *Candida parapsilosis* biofilm production, which adds to worldwide public health problems. The results of the study showed that the combination of curcumin and GO demonstrated a range of antibacterial activities, including antiplanktonic, antiadhesive, and antibiofilm capabilities. Cell viability dropped by 72% as a result of the therapy. After 72 h, the release of extracellular substances significantly decreased by 85%. These findings demonstrate that the combination of curcumin and GO offers a vital defense against microbial colonization of medical equipment. By mitigating the threat of biofilm-related infections, this approach addresses a critical concern in healthcare settings. In order to develop antibacterial coatings suited for diverse applications, Oves et al. [176] tested the effects of a composite system made of copper, curcumin, and GO on the contact killing of microbes and disinfection. Zone inhibition and scanning electron microscopy examinations of the nanocomposite, which was made into paintable coatings, showed antimicrobial characteristics through contact-killing processes. At a concentration of 25 µg/mL, they discovered that the nanocomposite exhibited the strongest antibacterial effects against *E. coli* with a zone of inhibition of 24 ± 0.50 mm and *Pseudomonas aeruginosa* with a zone of inhibition of 18 ± 0.25 mm. Additionally, it was found that the bactericidal concentration was 160 µg/mL and the minimal inhibitory concentration (MIC) was 80 µg/mL. This study concluded that when coated on various contact surfaces such as medical devices, the nanocomposite material exhibits outstanding antibacterial activity against *E. coli* and *P. aeruginosa*. This suggests that the likelihood of nosocomial infections, which are diseases acquired in healthcare settings, may be significantly reduced. In a study conducted by Shamsi and colleagues [177], the efficacy of gallic acid-loaded GO against several MRSA strains was investigated using various methods. The results showed that this nanocomposite has antibacterial activity equivalent to that of the drug cefoxitin, specifically against MRSA and methicillin-sensitive *S. aureus* (MSSA) strains at concentrations of less than 150 µg/mL. The nanocomposite also showed a considerably delayed response against both MRSA strains, especially in terms of enhanced inhibition after exposure over a duration of 8 to 24 h. Importantly, the outcomes showed that the nanocomposite was more potent than free natural gallic acid. According to this work, the gallic acid-loaded GO nanocomposite has potential antibacterial properties against MRSA strains. However, further research is necessary to acquire a greater understanding of the potential applications and mechanisms of action of this nanocomposite as a potential antibacterial agent. Another instance is the research by Arfat et al. [178], which generated antibacterial nanopackaging films by combining clove essential oil and GO nanosheets with PLA. *S. aureus* and *E. coli* germs were effectively combated by these films. With this novel technique, it may be possible to develop packaging materials that are effectively antibacterial and aid in maintaining the quality and safety of diverse items. In an earlier study, Liu et al. [179] incorporated the garlic-derived chemical allicin into composites of chitosan, polyvinyl alcohol, and GO to create nanofibrous membranes. The controlled release of allicin from the membrane provided this composite with both sustained releasing behavior and significant antibacterial activity. This system’s antibacterial properties were particularly efficient against *S. aureus*, indicating its potential utility in tissue engineering and antibacterial wound dressings, among other applications. Successful fabrication of a multifunctional covering for orthopedic implants was demonstrated by Xia-Ying et al. [180]. They used GO to apply berberine alkaloid to the surface of a biomedical titanium implant. The outcomes showed that this coating has extraordinary antibacterial properties, which were especially noticeable in its enhanced antibacterial activity against *S. aureus*. As opposed to the antibacterial activity of berberine alone, this improvement was due to the synergistic impact of GO and berberine. These results, which emphasize the potential of this coated implant to reduce bacterial infections after implantation, were verified by both in vitro and in vivo experiments. Ning et al. [181] also suggested a targeted strategy to prevent MRSA from forming biofilms. They used GO, functionalized it using an aptamer, and loaded it with berberine to create a bifunctional complex. A DNA aptamer known as PBP2 was coupled with berberine to create an aptamer/berberine bifunctional complex. Through π−π interactions, this complex was then adsorbed onto GO. By lowering cell-surface attachment, the approach drastically reduced MRSA biofilm development. Additionally, the simultaneous delivery of berberine and the PBP2a-targeted aptamer via the GO platform revealed efficient antibiofilm action. 

Undoubtedly, the existing findings collectively indicate that using natural therapeutic agents in combination with GO-based materials is a viable strategy to treat microbial diseases, including but not limited to bacterial infections. This new sector has significant promise to tackle problems with biofilm development and antibiotic resistance. Several important suggestions may be provided, as shown in Table 9, for further research and use of natural therapies with GO-based materials.

## 6. Bone Regeneration Systems

Recent developments in bone tissue engineering have been impressive, with an emphasis on creating solid connections between grafts and bone components. Osteoconduction and osseointegration are both aspects of connection that are essential for facilitating the repair and regeneration of damaged bone tissues [182]. Therefore, the improvement in bone tissue regeneration depends on the delivery of signaling molecules and the sustained release of bone growth factors. Here are a few instances that highlight the advantages of NGO composite scaffolds and provide an overview of the function of GO-based materials in this field of research. Shen et al. [183] evaluated the effects of adding NGO sheets to a photopolymerizable poly-D, L-lactic acid (PDLLA)/polyethylene glycol hydrogel in order to promote the long-term release of chondroinductive growth factor (TGF-3). Increased initial mechanical strength, sustained release of TGF-3 (for up to 4 weeks), high cell viability of human bone marrow-derived mesenchymal stem cells (hBMSCs), and higher expression of chondrogenic genes (including aggrecan, collagen type II, SOX9, and cartilage matrix production) as compared to GO-free scaffolds of TGF-3, are all made possible by the incorporation of sheets. In vivo experiments show that hBMSC-seeded TGF-3/PDLLA exhibits a much greater cartilage matrix than their TGF-3/PDLLA counterparts without GO. Bone morphogenetic protein-2 is continuously released through NGO-based administration, which affects the activation of the NF-B signal transduction system, as stated by Zhong et al. [184]. Effectively, bone morphogenetic protein 2 (BMP2) was adsorbed onto GO flakes with diameters ranging from 81.1 nm to 45,749.7 nm. The findings showed that GO continuously prolonged BMP2 release (for at least 40 days). The biocompatibility of chondrocytes or bone marrow stem cells was not significantly altered by BMP2-GO. Evaluated in vivo in a rat model of osteoarthritis also showed a considerable improvement, suggesting that GO may be able to regulate the release of this protein. They concluded that by promoting continuous BMP2 release, the BMP2-GO approach might reduce the activity of the NF-B pathway. The study carried out by La et al. [185] reported the GO delivery of substance P (SP) and BMP2 coated on titanium (Ti) implants. According to their research, the coated GO permits constant release of BMP2. It is anticipated that the combined delivery of BMP2 and SP will improve osteointegration of dental or orthopedic implants since it promotes bone growth on Ti placed in mice calvaria. 

Natural substances have significant potential as medicinal products because of their wide range of pharmacological and therapeutic effects. Researchers need to address issues with microbial infections, the possibility of malignant development, and other unfavorable consequences that might obstruct the scaffold’s successful integration inside the human body when it comes to bone production scaffolds and implants. The following paragraphs provide an overview of the literature on this particular study issue, despite the fact that it is currently limited. Faraji et al. [186] constructed an electrospun scaffold by electrospinning a nanofibrous scaffold (PCL/GO/Q) after first loading quercetin onto PCL. The scaffold’s minimal or insignificant toxicity toward cell development is a critical factor in bone repair. According to the results, PCL/GO/Q had a favorable effect on NIH/3T3 fibroblast cells, which demonstrated high cell viability (about 95%), proliferation, and adhesion. This shows that the material is favorable for cell development and wound healing. Additionally, even at low concentrations, the scaffolds showed noticeably improved antibacterial activities, which are crucial for avoiding bacterial infections. The use of artificial bone substitutes as a potential treatment for defects in bones led Guo et al. to develop a novel approach for treating bone defects with artificial bone substitutes [187]. They created a hybrid scaffold known as GO-BAI/DBM using GO nanosheets, flavonoid baicalin (BAI), and demineralized bone matrix (DBM). By steadily releasing the bioactive BAI via GO nanoparticles, this scaffold intends to promote bone repair. According to their findings, baicalin was released over a period of 14 days at a rate of 81.7%. Additionally, the scaffold had two effects: it increased bone marrow stromal cells’ ability to differentiate into osteoblasts and caused macrophages to take on a pro-healing M2 phenotype as a result of the baicalin released. The scaffold increased tissue integration and the inflammatory microenvironment after being implanted in rat subcutaneous tissue. The scaffold has most impressively shown remarkable abilities in fostering considerable bone repair in rats over a 12-week duration. These results show the possibility of scaffolds that combine natural molecules with diverse effects. Through the provision of new replacements with advantageous multifunctional properties, this creative technique shows potential for enhancing bone tissue engineering. An osteoinductive scaffold designed by Kashte et al. [188] used a naturally occurring bioactive substance to stimulate the production of osteoblasts. PCL-GO-CQ is the outcome of the layer-by-layer construction of this scaffold utilizing electrospun PCL, *Cissus quadrangularis* extract, and GO. According to their research, the synergistic effects of GO and plant extract increased the potential for osteoblastic differentiation, osteoconduction, and osteoinduction. This exciting combination has significant potential for use in tissue engineering and bone regeneration.

**Figure 8:**
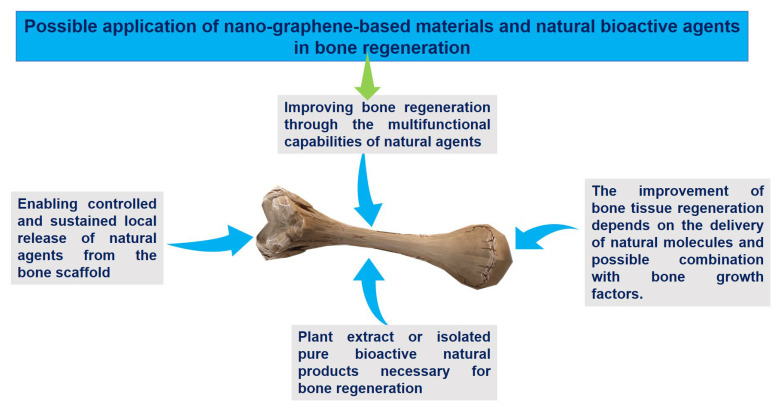
The advantages of using delivery systems for natural agents for bone regeneration filed.

Despite the previously conducted research, little is known about how NGO formulations combined with natural substances might improve bone regeneration and their use in daily life. When developing intelligent delivery systems for this particular utilization, researchers need to consider a number of important benefits and considerations (Figure 8 and Table 10).

## 7. Conclusions and Future Prospects

Over the years, materials based on graphene and NGO have demonstrated tremendous promise for use in biomedical applications. Although the initial focus of the research was mostly on cancer therapy, these materials have been shown to have a wide range of applications in tissue engineering, treatment of microbial infections, and a variety of biomedical and pharmaceutical settings. Their attractive physicochemical characteristics, which include a large surface area and a range of structural attributes (stability, mechanics, electrical properties, flexibility, and conductivity), fit well with biomedical demands. These materials have increased therapeutic agent efficacy, surface functionality, efficient drug carrier capability, high drug-loading capacity, biocompatibility, controlled drug release, bioimaging, and multifunctional nanoplatforms. Therefore, they are particularly suitable nanomaterials for the controlled release of drugs in a variety of disease therapies. This review provides an overview of the many surface modification techniques that are necessary to develop multifunctional systems that enhance biocompatibility, loading, release, and targeting efficiency of therapeutic medicines. It also reviews drug-loading and control methods used for NGO and GO-based materials, primarily anticancer medicines, highlighting their outstanding potential as carriers for efficient drug delivery. The final sections of this review provide insight into different approaches to delivery using natural therapeutic substances, including those for bone regeneration, bacterial infections, and cancer. To address the limited published evidence and establish a realistic approach for clinical therapy across a range of diseases, noteworthy ideas are offered to broaden insights into building pharmaceutical formulations integrating natural medicinal agents with NGO. In this context, we highlighted the advantages of employing NGO to deliver herbal remedies in the treatment of bacterial infections, cancer, and bone regeneration, providing exciting new research directions. NGO-based nanocarriers’ remarkable aqueous solubility enables the efficient distribution of naturally insoluble substances while maintaining their therapeutic potential. The problem of the restricted solubility of natural substances can be solved using this method. Additionally, NGO offers a perfect platform for combining several natural compounds to achieve synergistic effects. One major benefit is its ability to encapsulate and load a variety of compounds, each with different pharmacological activities, including alkaloids, flavonoids, phenolics, and essential oils. With the help of non-covalent interactions (such as π−π stacking, hydrogen bonding, and hydrophobic interactions), covalent bonding, and a mixture of both, the NGO’s high surface area facilitates effective medication administration. Additionally, using natural agents into scaffolds or implants has considerable potential in specialized sectors such as bone regeneration therapy. The scaffold’s NGO material allows these integrated natural therapeutic compounds to release gradually over a lengthy period of time (such as weeks). By simultaneously encouraging bone cell development and exerting antimicrobial properties to prevent infections in the early stages of implantation, for example, this dual action strategy can have a variety of advantages. Furthermore, it may have osteoinductive and antifibrosis effects, promoting bone repair following the first implantation. Through innovative techniques for delivery, potent monotherapy can be achieved by utilizing these latent therapeutic properties of natural medical products in combination with the NGO nanocarrier.

## Figures and Tables

**Table 1:** Major classes of plant-derived natural products with their chemical structures.

Class Type of Natural Compounds	Examples
**Alkaloids**	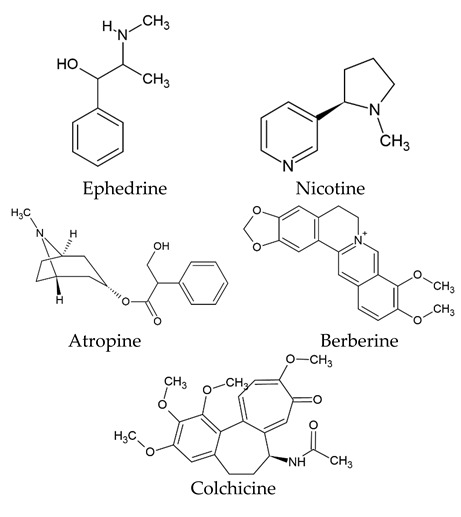
**Phenolic compounds**	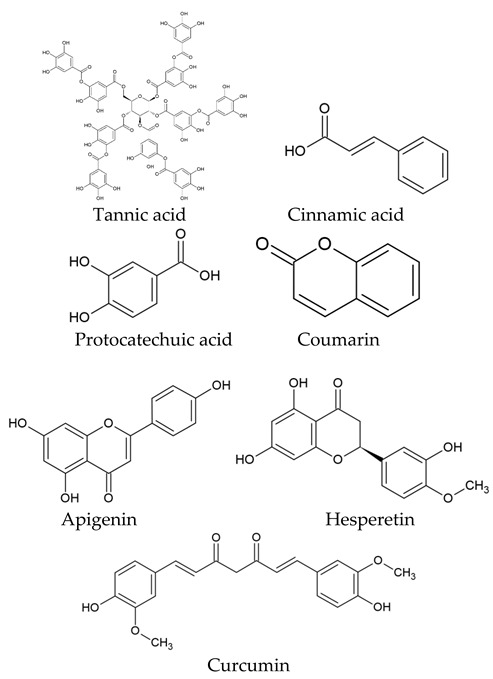
**Terpenoids**	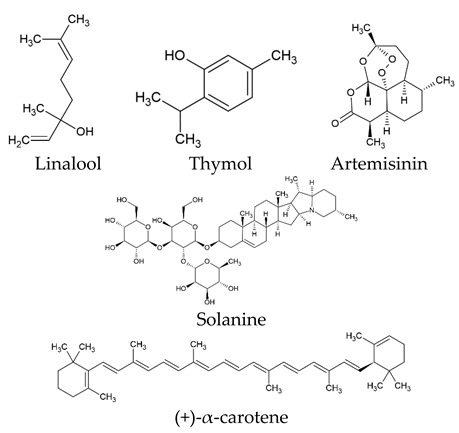
**Sulfur-containing compounds**	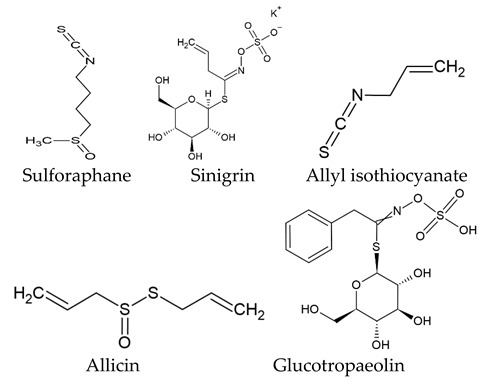

The chemical structures of these compounds were obtained from chemspider.com, the website of Royal Society of Chemistry (http://www.chemspider.com/Chemical-Structure, accessed on 21 September 2023).

**Table 2:** Plant-derived natural products approved for clinical applications and clinical trials in the treatment of various diseases [70,79].

Natural Agent	Natural Product Class	Plant Source	Trade Name/Year of the Introduction	Therapy
Vincristine	Vinca alkaloids	*Catharanthus roseus* L. *formerly Vinca rosea* L.	Vincristine/1963/FDA	Cancers
Artemisinin	Sesquiterpene lactone	*Artemisia annua*	Artemisinin/1987/FDA	Malaria
Arglabin	Sesquiterpene lactone	*Artemisia glabella*	Arglabin/1999/FDA	Cancer chemotherapy
Capsaicin	Alkaloid	*Casicum Annum* L.	Qutenza/2010/FDA	Post therapeutic neuralgia
Colchicine	Alkaloid	*Colichicum*	Colcrys/2009/FDA	Gout
Dronabinol/ Cannabidiol/Cannabinol	Alkaloid	*Cannabis Sativa* L.	Sativex/2005/FDA	Chronic neuropathic pain
Galanthiamine	Alkaloid	*Galanthus Cancasicus*	Razadyne/2001/FDA	Dementia associated with Alzheimer’s disease
Ingenol mebutate	Diterpene ingenol	*Euphorbia peplus* L.	Picato/2012/FDA	Actinic keratosis
Masoprocol	Phenolic lignan	*Larrea tridentata*	Actinex/1992/FDA	Cancer chemotherapy
Omacetaxine mepesuccinate (Homoharringtonine)	Alkaloid	*Cephalotaxus harringtonia*	Synribo/2012/FDA	Cancer
Paclitaxel	Alkaloid	*Taxus brevifolia* Nutt.	Taxol/1993/FDA Abraxanec/2005/FDA Nanoxelc/2007/FDA	Cancer chemotherapy
Solamargine	Alkaloid	*Solanum* spp.	Curadermd/1989	Cancer chemotherapy
Ingenol mebutate	Diterpenes	*Euphorbia peplus* L.	In clinical evaluations	Potent antiproliferative effects for cancers
Epigallocatechin-3-O-gallate (EGCG)	Polyphenolic	*Camellia sinensis* (L.)	In clinical evaluations	Antiviral, Alzheimer’s disease, cardiac amyloid light-chain amyloidosis, others
Curcumin	Polyphenolic	*Curcuma longa* L.	In clinical evaluations	Cancers, Alzheimer’s disease, fibromyalgia, cardiovascular disease
Genistein	Phenolic (flavonoid)	*Genista tinctoria* L.	In clinical evaluations	Cancers
Betulinic acid	Triterpene	*Gratiola officinalis* L.	In clinical evaluations	Cancers
Gossypol	Phenolic	*Gossypium* *hirsutum* L.	In clinical evaluations	Leukemia cancers
Quercetin	Phenolic (flavonoid)	*Allium cepa* L. and other plants	In clinical evaluations	Cancers, diabetes mellitus, obesity diastolic heart failure, hypertensive heart disease, Alzheimer’s disease, others
Resveratrol	Phenolic	*Vitis vinifera* L.	In clinical evaluations	Diabetes, vascular liver disease, cardiovascular disease, inflammation, insulin resistance, bone disease, coronary artery disease, obesity, oxidative stress, others

**Table 3:** Advantages and disadvantages of natural and synthetic drugs [80,81,82,83,84].

	Natural Drugs	Synthetic Drugs
**Advantages**	- Ease of access - Relative safety - Synergistic effects - Cultural acceptance - Long history of use - Traditional knowledge - Environmentally friendly - Complex chemical composition - Health benefits beyond treatment - Potential for novel drug discovery	- Cost-effectiveness - Potency and efficacy - Targeted drug design - Rapid drug development - Precision and consistency - Controlled side effect profile - Intellectual property protection - Improved stability and shelf life - Reduced contamination and allergenicity
**Disadvantages**	- Limited supply - Variable potency - Limited shelf life - Ethical considerations - Lack of standardization - Potential contamination - Risk of allergic reactions - Lack of rigorous clinical trials - Interaction with other medications - Standardization and regulatory challenges	- Drug resistance - Ethical concerns - Drug interactions - Environmental impact - Side effects and toxicity - Lack of natural synergy - Limited natural diversity - Patent exclusivity and cost - Development time and costs - Unforeseen long-term effects

**Table 4:** Summary of possible controlled responsive release of drugs from NGO.

Stimuli Classification	Stimuli Kind	Carrier	Drug/Loading	Release Mechanism	Ref.
**Internal-stimuli release**	pH-responsive release	Pluronic NGO-pluronic F127	Doxorubicin/loaded onto PF127/GO nanohybrid.	A change in pH can cause the release by changing hydrogen bonds and the solubility of the drug.	[118]
		NGO-sulfonic acid groups-folic acid	Doxorubicin and camptothecin are loaded onto this nanocarrier via π–π stacking and hydrophobic interactions.	These drugs were released from NGO into an aqueous solution as their hydrophilicity increased, making them more water-soluble and hydrophilic.	[119]
		NGO-PEG	Phenformin encapsulated onto NGO through hydrogen bonds and π–π stacking interaction	The drug release varies according to the shifting zeta potential of the prepared loaded material in the surrounding media. At acidic pH levels, improved positive phenformin release results from an electrostatic potential (at the shear planes of PNGS).	[120]
		NGO-PEG	Doxorubicin drug loaded through hydrogen bonding and π–π bonding	The release can take place due to the partial hydrogen bonds dissociation that connects NGO, DOX, and the -OH and -NH_2_ groups in a low acidic environment, thereby accelerating drug release.	[121]
	Redox-responsive release	NGO-SS-mPEG	Doxorubicin hydrochloride loaded via π–π bonding	Increasing the intracellular GSH concentrations leads to rapid drug release that may relate to drug diffusion from the carriers as well.	[122]
		NGO-PEG-NH_2_-RGD	Doxorubicin	With more GSH reduction, along with evaluated special photothermal performance, the response release happens.	[123]
		NGO-hyaluronic acid	Doxorubicin via π–π stacking and hydrophobic interactions onto NGO sheets	With the presence of GSH at various concentrations, the release accolated through thiol exchange from NGO’s surface.	[124]
	Temperature-responsive release	Poly(N,N-diethyl acrylamide)/ functionalized GQD-thermosensitive hydrogels	Doxorubicin	Drug releases from the nanocomposite at the range of 28–42 °C. The release takes place due to diffusion kinetics.	[125]
		NGO-functionalized polymer	Quercetin and 5-FU as hydrophobic and hydrophilic drugs.	These drugs release in response to changing temperature levels.	[126]
**External-stimuli release**	Magnetic-responsive release	Magnetic nanoparticles incorporated NGO-chitosan/alginate nanocomposites	Doxorubicin hydrochloride loaded into nanocomposite via π−π stacking and electrostatic attraction	It releases corresponding to magnetically stimulated effect and produces uptake.	[127]
		Polymeric-magnetic-GO	Doxorubicin	The release effect happens according to the presence of magnetic triggering.	[128]
	Light-responsive release	NGO-PEG	Doxorubicin	NIR and pH dual-responsive affects releasing of DOX loaded by noncovalent bonding modification.	[129]
		NGO-PEG	Photosensitizer molecule (Chlorin e6) loaded via non-covalent bonding as a photodynamic therapy	The photothermal effect promotes the delivery and release of Ce6 when exposed to a near-infrared laser.	[130]
**Combined-responsive release**	Combined-responsive release with dual or triple effect	Functionalized NGO-based materials	Many therapeutics	Drug molecules release depending on various conditions accelerating their release profiles to produce therapeutic action and cellular uptake.	[129,131,132]

**Table 5:** Summary of studies on the loading and release of NGO-based materials incorporating approved anticancer drugs.

Drug Carrier	Drug Model	Loading Method	Drug Loading	Drug Content	Drug Release	Ref.
NGO-polydopamine	Cytarabine hydrochloride-Hydroxycamptothecin	Non-covalent bonds	35% 43%	11.3% 19%	50% 50%	[103]
NGO-polydopamine conjugated	Methotrexate	Non-covalent bonds	81.88%	19.72%	80%	[90]
NGO-PEGylated	Doxorubicin	Physical adsorption	90%	10%	65%	[133]
NGO-conjugated FSHR antibody	Doxorubicin	Adsorption	75.6%	8.4%	69.3%	[134]
NGO-PEG	Doxorubicin/ Cisplatin	Combined method	36.7% 37.6%		64.6% 65.7%	[88]

**Table 6:** Summary of studies on loading and release of NGO-based materials incorporating natural agents for different diseases.

Drug Carrier	Drug Model	Drug Content	Drug Release	Application	Ref.
NGO-functionalized collagen scaffold	Curcumin	NA	82.5%	Antimicrobial and wound healing tissue engineering	[135]
NGO-PEG	Curcumin	4.5%	60%	NA	[136]
GO-liposome complex	Curcumin	NA	71.2	Antibacterial in topical disease	[137]
Folate-PEG-phospholipid coated RGO nano assembly (FA-PEG-Lip@rGO/Res)	Resveratrol	69.5 ± 4.3%	Up to 40.57%	Anticancer	[138]
NGO	Quercetin	Up to 35%	Negligible at 24 h	Anticancer	[139]
NGO-gelatin-polyvinylpyrrolidone (PVP) nanoemulsion	Quercetin	45%	91% and 95.5%	Anticancer	[140]
NGO-PVP	Essential oil	87.08%	NA	Anticancer	[141]
GO-chitosan nanocomposites	Proanthocyanidins (from grape seed extract)	Aprox. 20%	28.4% to 100%	NA	[142]

**Table 7:** Achieved active targeting ligands used in the development of targeted delivery systems using GO nanomaterials.

Ligands	Cancer Type	Drugs/Therapeutic Agents	Approach	Findings	References
HN-1 peptide	Oral squamous cell carcinoma (OSCC)	Doxorubicin drug	Through hydrogen and π–π bonds	Due to extensive tumor targeting, it causes higher cellular uptake and cytotoxicity in OSCC cells.	[145]
Fibroblast activation protein (FAP, a membrane-bound protease)	Oral squamous cell carcinoma (OSCC)	Doxorubicin drug	Via π–π stacking	It exhibits specific targeting effects for OSCC with improved tumor suppression performance in vivo and in vitro.	[121]
Tumor-specific antibody SCCA (8H11)	Squamous cell carcinoma	Cisplatin drug	Non-covalent adsorption	Attaching antibody demonstrates the capacity to target squamous cancer cells with efficient killing of cancer cells combined with limiting the toxicity to non-cancer cells. The data obtained from the nude mouse tumor-bearing model shows the new approach to therapy for squamous cell carcinoma to be both safe and effective.	[115]
Folic acid	Cancer cells	Doxorubicin drug	Covalent amide bond	It enhances receptor-mediated endocytosis, which helps the internalization of tumor cells. Additionally, it demonstrates a targeted chemo-photothermal therapy with good anticancer therapeutic effectiveness that precisely delivers medicine and heat to tumor sites.	[146]
Folic acid	Human cervical adenocarcinoma cell line	Chlorambucil drug	Covalent amide bond	More cytotoxic on cancer cells.	[147]
EGFR targeting GE11 peptide	Esophageal cancer cells	Oridonin natural agent	Covalent bonds	For esophageal cancers, the system exhibits a high ability to target cancer in combination with anticancer efficacy via the EGFR pathway.	[148]
Transferrin (Tf)	Murine mammary carcinoma cell line (EMT6)	Dihydroartemisinin (natural agent) and transferrin	Covalent bonds	When compared to the drug alone, the system significantly increases tumor delivery specificity and cytotoxicity. It also shows that it can substantially reduce tumor burden in mice while producing only minor side effects.	[149]
Folic acid	Breast cancer	Doxorubicin drug	Covalent bonds	Targeted delivery via FA-conjugated and loaded anticancer drug could be a safe and efficient treatment for breast cancer.	[150]
Monoclonal antibody (mAb) against follicle-stimulating hormone receptor (FSHR)	Metastatic breast cancer	Doxorubicin drug	Covalent bonds	This focused system demonstrates an effective tool for early metastasis selective killing in vivo animal model.	[134]
TRC105, a monoclonal antibody	Breast cancer	NA	Covalent bonds	A functionalized NGO with active ligands demonstrates specifically targeted cancer cells.	[151]
Folic acid	Ovarian cancer cells	miRNA (let-7i) combined platinum	Covalent bonds	The system shows effective action against cisplatin resistant SKOV3 cells.	[152]

**Table 8:** Active cancer targeting by combining GO-based materials and natural therapeutics and some remarks.

Ways and Characteristics of Active Cancer Targeting and Drug Loading	Remarks
NGO in the form of nanoparticles or nanosheets can connect an active targeting ligand on the surface via adsorption and chemical bonds.	Facilitating endocytosis mediation, cellular uptake, and internalization, and consequently enhancing the anticancer therapeutic effects.
The conjugation of ligands, e.g., aptamers, antibodies, and small molecules can be achieved before or after the natural agent’s therapeutic encapsulation/loading.	The way of conjugation “before, during, and after” does not affect either loading content or efficiency properties. It can be related to the ratio between loading and ligands conjugation.
It is important to know the solubility of natural agents and targeting ligands.	In case of loading well before the ligand’s conjugation, it is better to use vice versa solubility. For example, if a natural agent(s) soluble in water is first used, it is better to use solvent for ligand conjugation, which does not dissolve the initially loaded/encapsulated amounts. In the case where therapeutic agents are used with ligands at the same time, it is suitable to employ proper solvent for both.
Most of the chemical bonding can occur according to carboxylic acid groups of NGO.	Use the proper chemical coupling crosslinking such as EDC/NHS chemistry to form amide bonds.
To ensure cellular uptake by passive or active targeting, scanning electron microscopy can be employed without fluorescence molecules. In the case of fluorescence dye, confocal laser scanning microscopy can be performed.	The qualitative and quantitative analysis of cellular uptake can be realized.
It is necessary to investigate different cancer cells to evaluate the active cancer targeting.	The reason for this is the differences between overexpression of receptors that mediate intracellular uptake, and thereby the anticancer effect.
The conjugation of targeting ligand contents on the surface of NGO may affect drug release percentages and profiles.	It is expected that with a small amount, there is no big difference between nanoformulations developed for “passive” or “active” targeting. However, in the case of a high fraction presented on NGO, drug release can be influenced and extended release could be expected for that situation.

**Table 9:** Antimicrobial delivery strategies based on GO-based materials and natural therapeutics and some remarks.

Antibacterial Designs	Remarks
GO-based materials	▪ It includes GO, reduced GO, and composites (with metal, metal oxides, and polymeric composites). ▪ It shows potential to be employed as a new weapon in combating infectious diseases and multidrug-resistant bacteria. ▪ Therefore, GO-based materials are recommended as therapeutic nanomedicine platforms. ▪ It needs to consider some points for designing nanotherapeutic antimicrobial agents with GO-based materials such as surface modification and functionalization to obtain biocompatible material and improve antimicrobial efficiency.
GO materials have inherent antibacterial effects.	▪ Some evidence indicated that GO nanomaterials themselves have shown antibacterial properties due to, e.g., mechanical cutting of bacterial membranes.
Many fabricated systems have different mechanisms of actions.	▪ The mechanism of action could be related to permeating the bacterial nucleic acid and cytoplasmic membrane. ▪ Cell wall integrity loss, nucleic acid damage, and increased cell wall permeability.
The delivery systems compared to traditional therapeutic agents can result in high antibacterial activity and selectivity.	▪ It can suppress bacteria such as *S. aureus* > 99%. ▪ It shows an efficient effect against resistant microbial such as methicillin-resistant *S. aureus* (MRSA).
Efficient killing of biofilm bacterial formation on medical devices and different approaches like antibacterial coating	▪ Combining GO nanomaterial and natural therapeutic agents can combat bacterial formation (by some strains including *Candida parapsilosis*, *S. aureus*), presenting a promising strategy for the main challenges in healthcare-associated infections. ▪ It can be used for orthopedic implants, wound dressing, and tissue engineering.
Disinfection and killing of microorganisms can perform, e.g., nanocomposites coatings.	▪ There are many approaches for this strategy, such as paintable coatings that exhibit antimicrobial properties by contact-killing mechanisms. ▪ It provides a significant reduction in the risk of nosocomial infection in hospitals.
Targeting microbial infection and biofilm formation by delivering therapeutics	▪ It can use ligands specifically for targeting the inhibition of biofilm formation such as methicillin-resistant *S. aureus*, generating effective delivery and therapy.

**Table 10:** Bone regeneration by combining GO-based materials and natural therapeutics and some remarks.

Advantages	Remarks
Improving bone regeneration through the multifunctional capabilities of natural agents	▪ It is important to take into account the proportion of natural agents within the scaffold or implant to ensure optimal cell viability, proliferation, and cell adhesion, which are vital for successful bone regeneration.
Enabling controlled and sustained local release of natural agents from the bone scaffold	▪ The effect of the prolonged release (days and weeks) should be considered since bone growth formation requires a long time.
Plant extract or isolated pure bioactive natural products necessary for bone regeneration	▪ A proper plant extract containing specific natural molecules with various pharmacological actions or pure compounds can be investigated. ▪ Both therapeutic agents could enhance the effects, i.e., osteoblastic differentiation, osteoconduction, and osteoinduction potential, which is highly likely in bone regeneration and tissue engineering applications.

## Data Availability

No data were created in this review.
